# Supplementary material for: Simultaneous Study of Anti-Ferroptosis and Antioxidant Mechanisms of Butein and (S)-Butin
Source: Molecules. 2020 Feb 5;25(3):674. doi: 10.3390/molecules25030674 (PMC7036861; doi:10.3390/molecules25030674)
Supplement: Supplementary file 1 [file molecules-25-00674-s001.zip › Suppls/Suppl. 1 Dose response curves inthe antioxidant spectrophotometric analyses.docx]

Supplementary File 1. Dose response curves in antioxidant colorimetric assays

Simultaneous Study of Antioxidant and Anti-ferroptosis Mechanisms of butein and (*S*) butin

Jie Liu^1^, Xican Li^2,^*, Rongxin Cai^2^, Ziwei Ren^2^, Aizhen Zhang^2^, Fangdan Deng^2^, Dongfeng Chen^3,4,^*

^1^ Shenzhen Bao'an Traditional Chinese Medicine Hospital, Guangzhou University of Chinese Medicine, Shenzhen 518000, China.

^2^ School of Chinese Herbal Medicine, Guangzhou University of Chinese Medicine, Waihuan East Road No. 232, Guangzhou Higher Education Mega Center, Guangzhou 510006, China

^3^ School of Basic Medical Science, Guangzhou University of Chinese Medicine, Guangzhou, China, 510006

^4^ The Research Center of Basic Integrative Medicine, Guangzhou University of Chinese Medicine, Guangzhou, China, 510006. E-mail: chen888@gzucm.edu.cn (D. C.)

* Correspondence: lixc@gzucm.edu.cn (X.L.); chen888@gzucm.edu.cn (D.C.)

1. linoleic acid emulsion assay

Figure S1: The dose response curves of butein and (S) butin in linoleic acid emulsion assay. Each value is expressed as mean ± SD (n = 3).

Tab. S1 The comparison of IC_50_ values of butein and (S) butin and positive control in linoleic acid emulsion assay.

|  | Mean ± SD (μg/mL) | Mean ± SD (mM) |
| --- | --- | --- |
| Trolox | 0.2 ± 0.3 | 1.0 ± 1.5 ^b^ |
| butein | 0.8 ± 0.3 | 3.2 ± 1.4 ^a^ |
| (S) butin | 12.7 ± 2.1 | 46.7 ± 7.4 ^b^ |

IC_50_ value was defined as the concentration of 50% superoxide anion radical inhibition and calculated by linear regression which was analyzed by Origin 6.0 professional software. Means values with different superscripts in the same column are significantly different (p<0.05).

1. Cu^2+^-reducing assay

Figure S2: The dose response curves of butein and (S) butin in Cu^2+^-reducing assay. Each value is expressed as mean ± SD (n = 3).

Tab. S2 The comparison of IC_50_ values of butein and (S) butin and positive control in Cu^2+^-reducing assay.

|  | Mean ± SD (μg/mL) | Mean ± SD (mM) |
| --- | --- | --- |
| Trolox | 22.9 ± 0.9 | 91.6 ± 3.8 ^b^ |
| Ascorbic acid | 14.7 ± 0.2 | 83.2 ± 1.2 ^b^ |
| butein | 9.8 ± 0.1 | 36.2 ± 0.1 ^a^ |
| (S) butin | 12.1 ± 0.5 | 44.4 ± 1.9 ^a^ |

IC_50_ value was defined as the concentration of 50% superoxide anion radical inhibition and calculated by linear regression which was analyzed by Origin 6.0 professional software. Means values with different superscripts in the same column are significantly different (p<0.05).

1. Fe^3+^-reducing assay

Figure S3: The dose response curves of butein and (S) butin in Fe^3+^-reducing assay. Each value is expressed as mean ± SD (n = 3).

Tab. S3 The comparison of IC_50_ values of butein and (S) butin and positive control in Fe^3+^-reducing assay.

|  | Mean ± SD (μg/mL) | Mean ± SD (mM) |
| --- | --- | --- |
| Trolox | 4.4± 0.1 | 8.7 ± 0.2 ^b^ |
| Ascorbic acid | 1.6 ± 0.1 | 4.5 ± 0.2 ^a^ |
| butein | 2.8± 0.1 | 5.3 ± 0.1 ^a^ |
| butein(S) | 3.1 ± 0.1 | 5.6 ± 0.1 ^a^ |

IC_50_ value was defined as the concentration of 50% superoxide anion radical inhibition and calculated by linear regression which was analyzed by Origin 6.0 professional software. Means values with different superscripts in the same column are significantly different (p<0.05).

1. PTIO·-scavenging assay

Figure S3: The dose response curves of butein and (S) butin in PTIO**^•^**-scavenging assay. Each value is expressed as mean ± SD (n = 3).

Tab. S4-1 The comparison of IC_50_ values of butein and (S) butin and positive control in PTIO**^•^**-scavenging assay (**pH= 4.5**).

|  | Mean ± SD (μg/mL) | Mean ± SD (mM) |
| --- | --- | --- |
| Trolox | 2.3 ± 0.1 | 9.1 ± 0.2 ^a^ |
| Ascorbic acid | 1.4 ± 0.1 | 8.2 ± 0.2 ^a^ |
| butein | 4.5 ± 0.1 | 16.4 ± 0.9 ^b^ |
| (S) butin | 5.0 ± 0.1 | 18.4 ± 0.4 ^b^ |

Tab. S4-2 The comparison of IC_50_ values of butein and (S) butin and positive control in PTIO**^•^**-scavenging assay (pH= 6.0).

|  | Mean ± SD (μg/mL) | Mean ± SD (mM) |
| --- | --- | --- |
| Trolox | 2.7 ± 0.1 | 11.1 ± 0.5 ^a^ |
| Ascorbic acid | 1.4 ± 0.1 | 8.3 ± 0.5 ^a^ |
| butein | 2.5 ± 0.1 | 9.3 ± 0.9 ^a^ |
| (S) butin | 12.9 ± 0.1 | 47.6 ± 7.1 ^b^ |

Tab. S4-3 The comparison of IC_50_ values of butein and (S) butin and positive control in PTIO**^•^**-scavenging assay (pH= 7.4).

|  | Mean ± SD (μg/mL) | Mean ± SD (mM) |
| --- | --- | --- |
| Trolox | 1.3 ± 0.2 | 5.2 ± 2.8 ^a^ |
| Ascorbic acid | 0.8 ± 0.1 | 4.7 ± 1.1 ^a^ |
| butein | 3.5 ± 0.1 | 12.9 ± 0.4 ^b^ |
| (S) butin | 4.7 ± 0.1 | 17.4 ± 0.4 ^b^ |

IC_50_ value was defined as the concentration of 50% superoxide anion radical inhibition and calculated by linear regression which was analyzed by Origin 6.0 professional software. Means values with different superscripts in the same column are significantly different (p<0.05).

1. DPPH**^•^**-scavenging assay

Figure S5: The dose response curves of butein and (S) butin in DPPH**^•^**-scavenging assay. Each value is expressed as mean ± SD (n = 3).

Tab. S5 The comparison of IC_50_ values of butein and (S) butin and positive control in DPPH**^•^**-scavenging assay.

|  | Mean ± SD (μg/mL) | Mean ± SD (mM) |
| --- | --- | --- |
| Trolox | 5.8 ± 0.2 | 23.3 ± 0.7 ^b^ |
| Ascorbic acid | 4.7 ± 0.1 | 27.0 ± 0.2 ^b^ |
| butein | 4.3 ± 0.1 | 15.8 ± 0.5 ^a^ |
| (S) butin | 8.1 ± 0.1 | 29.9 ± 0.5 ^b^ |

IC_50_ value was defined as the concentration of 50% superoxide anion radical inhibition and calculated by linear regression which was analyzed by Origin 6.0 professional software. Means values with different superscripts in the same column are significantly different (p<0.05).
