# Supplementary material for: Simultaneous Study of Anti-Ferroptosis and Antioxidant Mechanisms of Butein and (S)-Butin
Source: Molecules. 2020 Feb 5;25(3):674. doi: 10.3390/molecules25030674 (PMC7036861; doi:10.3390/molecules25030674)
Supplement: Supplementary file 1 [file molecules-25-00674-s001.zip › Suppls/Suppl. 2 MS elucidation of standard butein and (S)-butin.docx]

Supplementary File 2. Original data of UPLC-ESI-Q-TOF-MS analysis for butein and (S) butin

Simultaneous Study of Antioxidant and Anti-ferroptosis Mechanisms of Butein and (*S*) butin

Jie Liu^1^, Xican Li^2,^*, Rongxin Cai^2^, Ziwei Ren^2^, Aizhen Zhang^2^, Fangdan Deng^2^, Dongfeng Chen^3,4,^*

^1^ Shenzhen Bao'an Traditional Chinese Medicine Hospital, Guangzhou University of Chinese Medicine, Shenzhen 518000, China.

^2^ School of Chinese Herbal Medicine, Guangzhou University of Chinese Medicine, Waihuan East Road No. 232, Guangzhou Higher Education Mega Center, Guangzhou 510006, China

^3^ School of Basic Medical Science, Guangzhou University of Chinese Medicine, Guangzhou, China, 510006

^4^ The Research Center of Basic Integrative Medicine, Guangzhou University of Chinese Medicine, Guangzhou, China, 510006. E-mail: chen888@gzucm.edu.cn (D. C.)

* Correspondence: lixc@gzucm.edu.cn (X.L.); chen888@gzucm.edu.cn (D.C.)

Fig.S1.1 Chromatogram of the butein when the formula [C_15_H_12_O_5_-H]^-^ was extracted

Fig.S1.2 primary MS spectra of the butein

Fig.S1.3 secondary MS spectra of the butein

Fig.S2.1 Chromatogram of (S) butin when the formula [C_15_H_12_O_5_-H]^-^ was extracted

Fig.S2.2 primary MS spectra of (S) butin

Fig.S2.3 secondary MS spectra of (S) butin
