# Supplementary material for: Simultaneous Study of Anti-Ferroptosis and Antioxidant Mechanisms of Butein and (S)-Butin
Source: Molecules. 2020 Feb 5;25(3):674. doi: 10.3390/molecules25030674 (PMC7036861; doi:10.3390/molecules25030674)

成都普瑞法科技开发有限公司
Chengdu Biopurify Phytochemicals Ltd.

Add: No.11 Building, No. 388 Rongtaidadao CNSTP
Wenjiang Zone, Chengdu, Sichuan, 611130 China
TEL: 028-82633987 FAX: 028-82633165
E-mail: biopurify@gmail.com sales@biopurify.com
Web: www.biopurify.com

Certificate of Analysis

Structure:

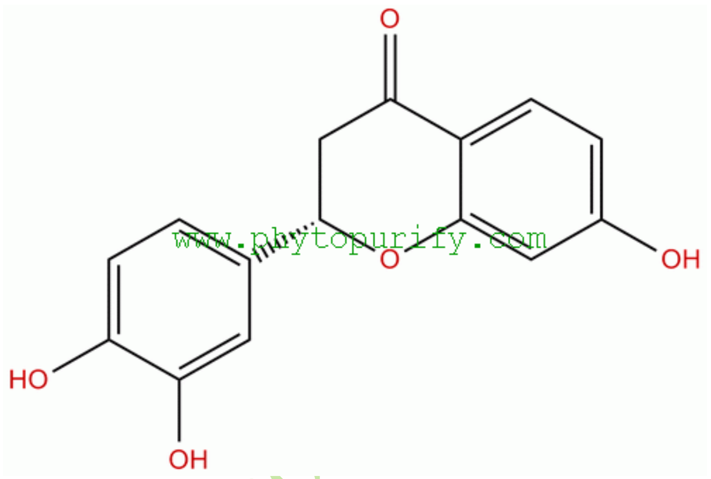

Product Name: Butin

Other Name:

Catalogue No.: BP0074

Batch No.: 14100903

Report date: 2014-10-09

CAS Number: 492-14-8

Mol. Formula: $C_{15}H_{12}O_5$

Mol. Weight: 272.256

Type of compound:

Identification Method: Mass, NMR

Analysis Method of Purity: HPLC-DAD

www.biopurify.com

Analytical result:

Test	Specification	Results
Appearance	Yellow powder	Yellow powder
Loss on drying	<3.0%	1.3 %
Purity (HPLC)	≥95.0%	97%

Package: Brown vial or HDPE Plastic Bottle

Storage: Cool and Dry place, protected from light, keep package airproofed when not in use.

Expiration: two years (2016-10-08) under conditions list above.

QC: Meng Pan **QA:** Lianglei Zhang

Date: 2014-10-09 **Date:** 2014-10-09

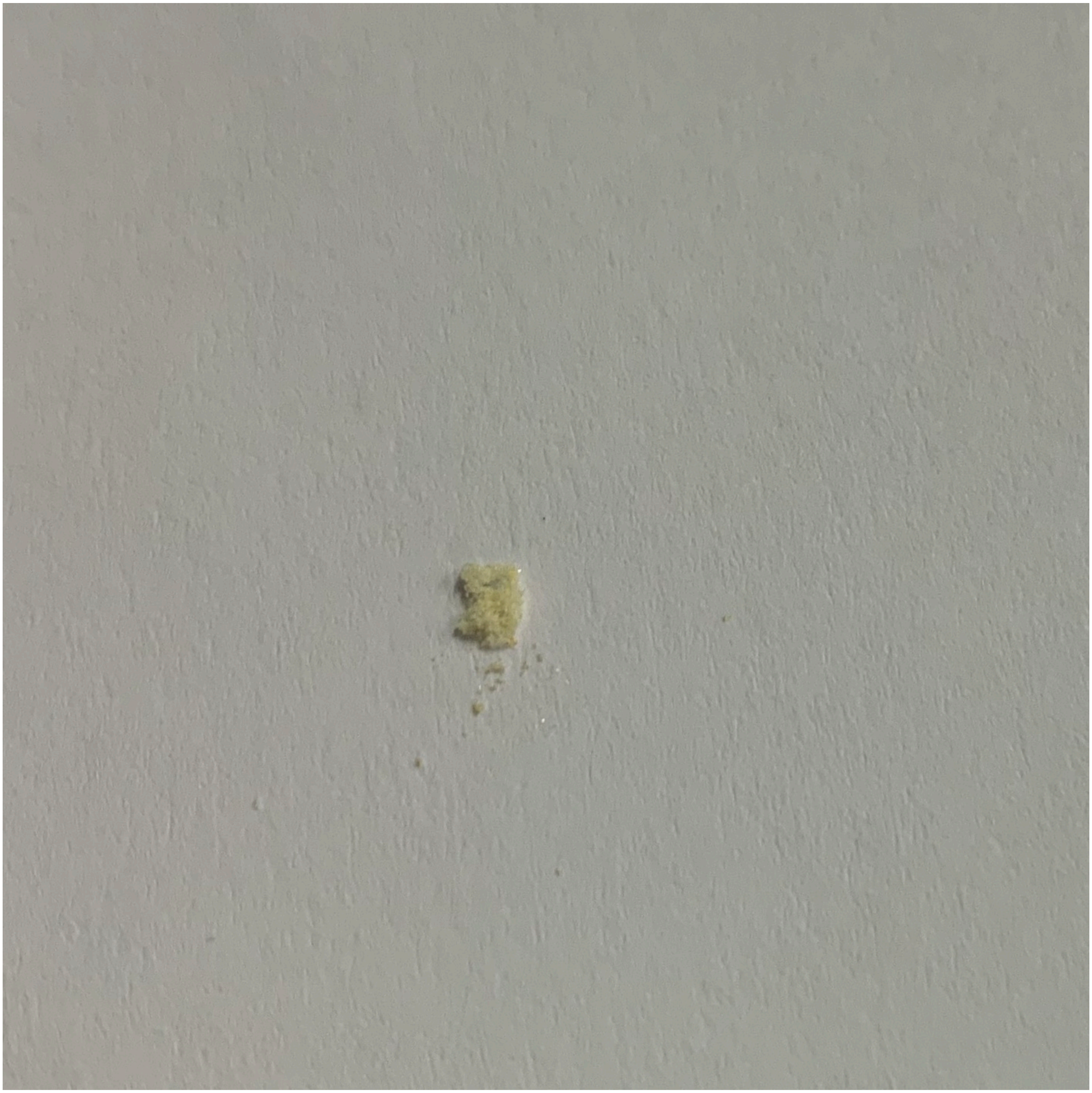

Supplement: Supplementary file 1 [file molecules-25-00674-s001.zip › Suppls/Suppl. 4 Certificate analysis of (S) butin.pdf]
