# Supplementary material for: Simultaneous Study of Anti-Ferroptosis and Antioxidant Mechanisms of Butein and (S)-Butin
Source: Molecules. 2020 Feb 5;25(3):674. doi: 10.3390/molecules25030674 (PMC7036861; doi:10.3390/molecules25030674)
Supplement: Supplementary file 1 [file molecules-25-00674-s001.zip › Suppls/Suppl. 5 Certificate analysis of butein.pdf]

产品分析证书
Certificate of Analysis

中文名称：紫铆因

English Name：Butein
别名(Alias):

产品编码(Cat. No.):SBP01739
CAS Number: 487-52-5
分子式(M. F.): C15H12O5
分子量(M. W.): 272.256

批号(Batch No.): PRF8042424
报告日期(Report date): 2017-04-24

Structure

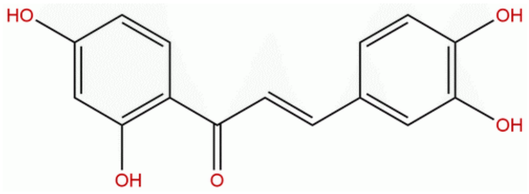

检验结果（Analytical result）：

检验项目（Test Item）	检验指标（Specifications）	检验结果（Results）
外观Appearance	Yellow powder	Yellow powder
干燥失重Loss on drying	＜3.0%	1.08%
纯度Purity (HPLC-DAD,380nm)*	≥98.0%	99.96%
质谱Mass	272.2±1	Conforms
核磁NMR	Comply with the structure	Conforms

* 色谱图见附件（**Please find HPLC chromatography attached.**）

检测方法（Test Method）： **Column:** Agilet 5TC-C18，4.6*150mm; **Column temperature**: 30℃; **Detection Mode:** UV380nm;**Flow Rate:** 1.0ml/min;

Sample dissolution: Methanol; **Mobile Phase: A,** 0.1% Phosphoric acid in water **B,** Acetonitrile;**Gradient elution:** B, 20%-40%15min

贮存条件（Storage）:2~8℃, protected from light, keep package airproofed when not in use.

复测期（Retest date）:two years (2019-04-23) under conditions list above.

QC: Zhang Ling　　　　QA: Wu Qi

Date: 2017-04-24　　　　Date: 2017-04-24

备注(Remarks): The sample solutions should be prepared and used on the same day, it is the best preparing the solutions immediately before use. If the solutons have to be made up in advance, it should be made as aliquots in tightly sealed vials at less than -20℃. Generally, these might be useable for up to two weeks.
In case of quality issue, please contact us within 15 days after receipt of the product.

Tel：+86-28-82633397　　Fax：+86-28-82633165
http://www.phytopurify.com　　Email：sales@biopurify.com　　biopurify@gmail.com

SAMPLE INFORMATION

Sample Name:	Butein	Acquired By:	System
Sample Type:	Standard	Sample Set Name:	
Vial:	27	Acq. Method Set:	Butein
Injection #:	1	Processing Method:	Samples
Injection Volume:	10.00 ul	Channel Name:	380.0nm
Run Time:	25.0 Minutes	Proc. Chnl. Descr.:	PDA 380.0 nm
Date Acquired:	2017-4-24 16:36:24 CST		
Date Processed:	2017-4-24 17:02:44 CST		

Auto-Scaled Chromatogram

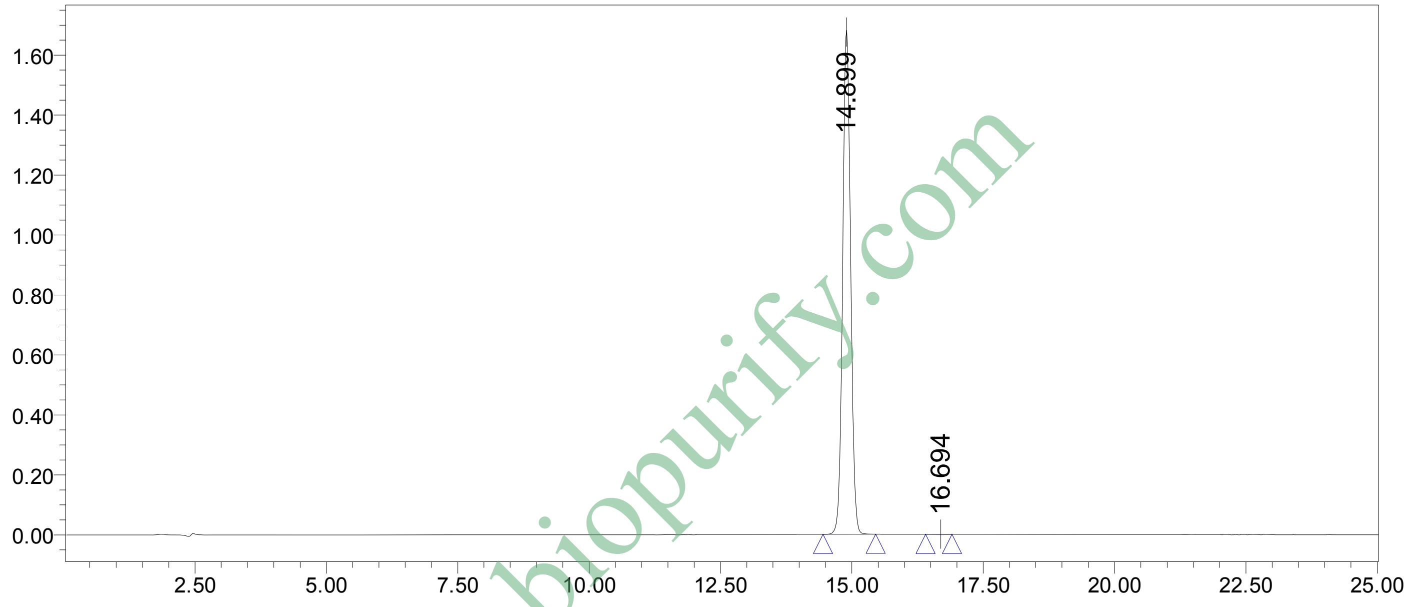

www.biopurify.com

Peak Results

	RT	Area	% Area	USP Plate Count	USP Resolution
1	14.899	17657289	99.96	46510.35	
2	16.694	7344	0.04	22624.64	5.95

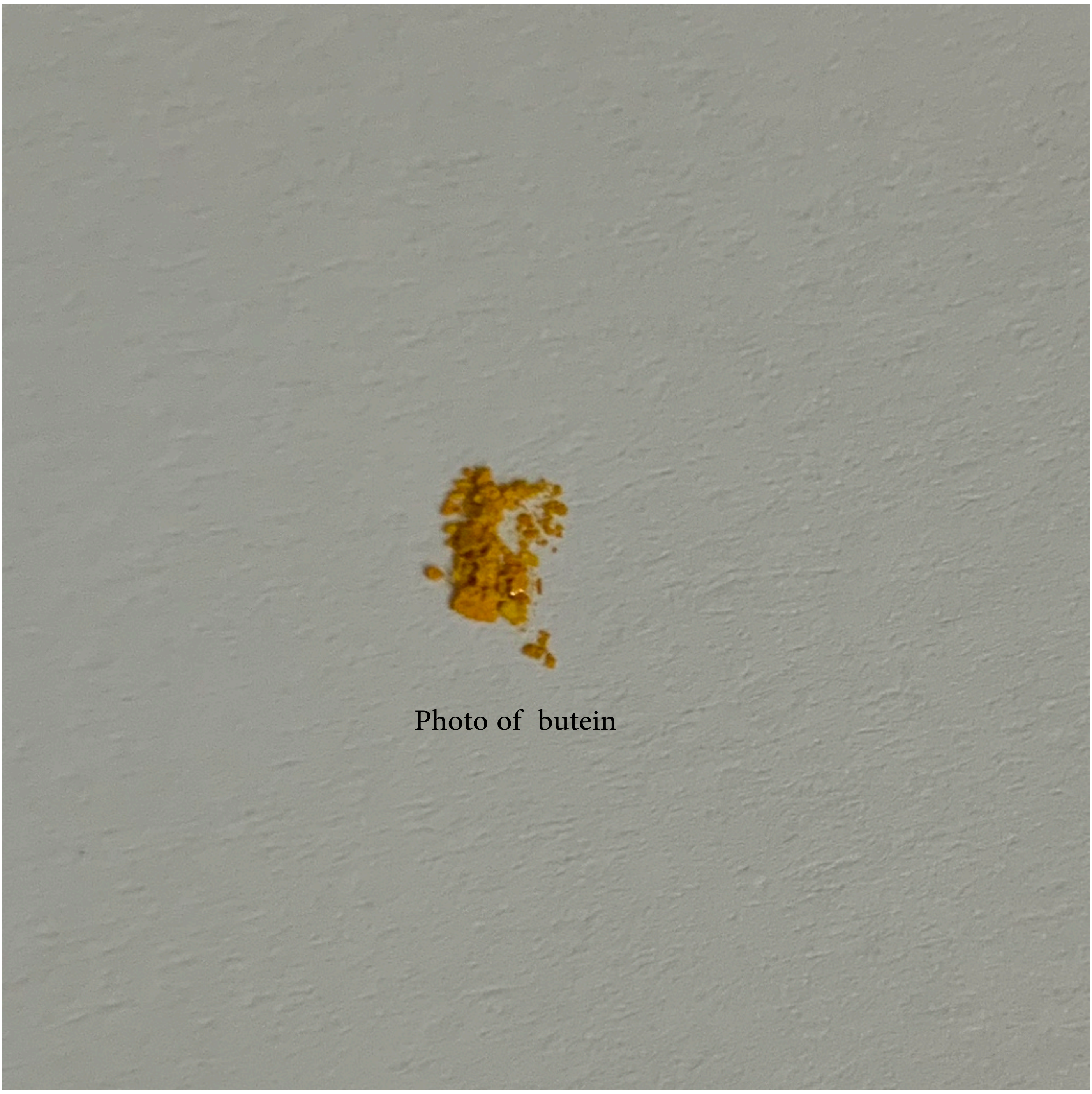

Photo of butein
